# Zinc-Finger 5 Is an Activation Domain in the *Saccharomyces cerevisiae* Stress-Responsive Transcription Factor Fzf1

**DOI:** 10.3390/jof12010015

**Published:** 2025-12-25

**Authors:** Ying Du, Wayne Y. Wang, Wei Xiao

**Affiliations:** Department of Biochemistry, Microbiology and Immunology, University of Saskatchewan, Saskatoon, SK S7N 5E5, Canada; yid154@usask.ca(Y.D.); w.wang@usask.ca (W.Y.W.)

**Keywords:** *Saccharomyces cerevisiae*, Fzf1, transcription regulation, zinc finger, chemical stress, transactivation domain

## Abstract

Fzf1 is a *Saccharomyces cerevisiae* transcription factor that contains five zinc finger domains (ZF1-5) and induces the expression of at least five genes in response to various chemical stresses by recognizing the shared promoter consensus sequence CS2. The N-terminal ZF1-3 are required and sufficient for binding to CS2, while ZF4 negatively regulates the activity of Fzf1. However, the effect of ZF5 on the activity of Fzf1 is not well defined. In this study, substitutions of the two zinc-coordinating Cys residues (C248S and C253S) of ZF5, or deletion of the whole ZF5 domain, compromised the chemical stress-induced activation of Fzf1. Since the elevated Fzf1-regulated gene expression caused by *fzf1-ZF4* could also be reversed by additional deletion of ZF5 or C248S/C253S substitutions, *fzf1-ZF5* mutations are epistatic over *fzf1-ZF4* mutations. Furthermore, *fzf1-ZF5* mutations are recessive to *FZF1*, while ZF5 is dispensable for the CS2 binding. Finally, Fzf1-ZF5 is required and sufficient to serve as a transcription activation domain when fused to a Gal4 DNA-binding domain. These observations collectively support a working model in which Fzf1 bound to its target gene promoters remains inactive due to an inhibitory activity of ZF4. Upon chemical stress, ZF4 is no longer able to inhibit the ZF5 transactivation activity, leading to the induction of Fzf1-regulated gene expression and subsequent chemical detoxification.

## 1. Introduction

*Saccharomyces cerevisiae FZF1* encodes a transcription factor that contains five zinc-finger (ZF) domains [1] (Supplementary Figure S1), and its highly conserved homologs can be found in lower eukaryotes, including but not limited to *Saccharomyces*, *Candida*, and *Kluyveromyces*, but are absent in higher eukaryotes. Fzf1 orchestrates expressions of target genes including *SSU1*, *YHB1*, *DDI2*, *DDI3* (*DDI2/3*), and *YNR064C* [2,3]. These genes are known for their response to a variety of chemical stimuli with a common theme of chemical stress tolerance. *SSU1* encodes a plasma membrane protein responsible for sulfite efflux [4]. *YHB1* encodes a dioxygenase participating in oxidative stress responses, particularly those mediated by nitric oxide (NO) [5]. It is worth noting that *YHB1* expression can be induced by NO and NO-derived compounds such as dipropylenetriamine (DPTA) NONOate, a commonly used experimental NO donor [6]. *DDI2/3* encode cyanamide (CY) hydratases [7,8] and can be induced by CY [8,9] or methyl methanesulfonate (MMS) [10], a DNA-damaging agent and potential methyl donor to macromolecules [11]. *YNR064C* encodes an epoxide hydrolase [12]. Overexpression of *FZF1* led to the induction of all five Fzf1-regulated genes [2], while deletion of *FZF1* abolished the chemical induction of these genes [3,13]. These observations collectively highlight the role of *FZF1* as a positive regulator for its downstream gene expression. It has been suggested by sequencing alignment and subsequently demonstrated that a conserved promoter element, named as consensus sequence 2 (CS2, 5′-AAA**TGATAG**TNAN**C**-3′, where “N” can be any nucleotides) present in all five Fzf1-regulated genes, is required for chemically induced *YHB1* and *DDI2/3* expression [2,13], highlighting the critical regulatory role of this sequence. Furthermore, in vitro and in vivo assays confirmed that Fzf1 directly binds the CS2 sequence from *DDI2/3* promoters [13] and other CS2 sequences [14], establishing CS2 as the specific DNA-binding target of Fzf1.

All five ZFs in Fzf1 belong to the Cys_2_His_2_ (C_2_H_2_) zinc finger family [15], which typically coordinate a tetrahedral zinc ion through two Cys and two His residues, positioning an α-helix into the major groove of duplex DNA [16]. Traditionally, each C_2_H_2_ zinc finger is thought to recognize a triplet of nucleotides [17]; however, Fzf1 has been characterized as a noncanonical C_2_H_2_ zinc finger protein [14]. A previous in vitro study [18] demonstrated that the N-terminal three zinc fingers (ZF1-3) are primarily responsible for recognizing the target DNA sequence in the *SSU1* promoter [18]. A recent CS2-bound Fzf1 crystal structural analysis [14] revealed that ZF1-3 is required and sufficient to recognize CS2. Since ZF4 and ZF5 appear to be dispensable for the CS2 binding, we proposed that they function as regulatory elements within Fzf1. Indeed, some previous studies [19,20] found that several single amino acid substitutions within Fzf1-ZF4 activated its selected downstream genes, and our recent studies demonstrated that ZF4 indeed serves as a repressor domain within Fzf1 [3], suggesting that ZF4 inhibits the Fzf1 activity in the absence of stimuli, and that the chemical induction is actually achieved through derepression.

When ZF4 is mutated or deleted, Fzf1 remains constitutively active, as judged by increased basal level expression of its target genes. Which element within Fzf1 is required to support such an activity? During our characterization of Fzf1-ZF4 functions, we observed that Fzf1 ZF1-3 alone, although capable of binding CS2, failed to activate its target gene despite chemical treatments [3], raising the possibility that ZF5, or other deleted residues in the construct, functions as an activator. Consistently, two Cys-to-Ser substitutions within the ZF5 domain abolished NO-induced *YHB1* expression [20]. Based on these observations, we hypothesized that ZF5 functions as a transcription activation domain (TAD) in Fzf1. To test this hypothesis, we created deletions and corresponding amino acid substitutions within Fzf1-ZF5 and assessed their phenotypes. Furthermore, Fzf1 and its various truncations were fused to a heterologous Gal4 DNA-binding domain (Gal4_BD_), and their transactivation activity was measured by reporter gene assays. These studies collectively demonstrated that ZF5 is required and sufficient to function as an activation domain.

## 2. Materials and Methods

### 2.1. Yeast Strains, Cell Culture, and Chemical Treatments

All haploid yeast strains used in this study were derived from BY4741 (*MATa his3Δ1 leu2Δ0 met15Δ0 ura3Δ0*). The isogenic *fzf1Δ::KanMX4* strain utilized in this study was from the yeast gene deletion collection. Yeast cells were cultured in yeast extract-peptone-dextrose (YPD) medium or synthetic dextrose (SD) medium supplemented with appropriate nutrients, as previously described [21].

For the chemical treatments, yeast cells were cultured in YPD or SD selective medium at 30 °C overnight. Then the cultures were inoculated into a fresh medium and grown for approximately 2 h until reaching an optical density of 0.2–0.3 at 600 nm (OD_600_). Cells were then treated with optimal concentrations of test chemicals, including 20 mM CY, 0.05% MMS, or 5 mM sodium sulfite supplemented with 75 mM tartaric acid (TA), for 2 h. For the NO treatment, DPTA NONOate was freshly dissolved in double-distilled water (ddH_2_O) to prepare a 0.1 M stock solution immediately before adding it to the culture medium, and cells were treated for 1.5 h. Untreated control cells were incubated under identical conditions.

### 2.2. Plasmid Construction and Site-Specific Mutagenesis

The YCpL-*FZF1* plasmid utilized in this study was as described previously [3]. Desired *fzf1-ZF5* mutations, including *fzf1*-*C248S*, *fzf1*-*C253S*, *fzf1*-*C157S,* *C248S* and *fzf1*-*C157S*, *C253S* (Supplementary Table S1), were generated by site-directed mutagenesis following a modified QuickChange protocol [22], using YCpL-*FZF1* and YCpL-*fzf1-C157S* as templates. Mutations were generated by PCR amplification of the plasmid using corresponding mutagenic primers (Table S2), followed by *Dpn*I digestion to remove the methylated template DNA. The resulting PCR products were transformed into *Escherichia coli* DH10B cells for selection of clones containing desired mutations. To make Fzf1 truncations in plasmid YCpL-*FZF1*, the same QuickChange protocol was followed except using overlapping truncation primers (Table S2). All *FZF1-*related mutations or truncations were confirmed by DNA sequencing.

### 2.3. Yeast Cell Transformation

Yeast cells were transformed with plasmids using a lithium acetate method [23] as described [24].

### 2.4. Yeast Survival Assay

Sulfite resistance was assessed by a serial dilution assay as previously described [25]. Plates containing 75 mM TA and different concentrations of sodium sulfite were prepared as described [3].

### 2.5. Yeast RNA Extraction and Quantitative Reverse Transcription PCR (qRT-PCR)

After chemical treatments, cells were harvested by centrifugation and subjected to enzymatic lysis by using 200 U of zymolyase (Amsbio, Cambridge, MA, USA, Cat. 120491-1) per 5 × 10^7^ yeast cells for 1 h. Total RNA was extracted from yeast cells using the Yeast RNA Extraction kit (Geneaid, New Taipei City, Taiwan, Cat. RBY300). The extracted RNA samples were then reverse transcribed into cDNA for long-term use, and gene expression levels were quantified by quantitative PCR with iQ™ SYBR Green Supermix (Bio-Rad, Mississauga, ON, Canada, Cat. 170-8882). Data analysis was performed using the 2^−ΔΔCT^ method as described [26] to determine relative expression levels of target genes normalized to the internal reference gene *UBC6*, as previously validated [13]. All experiments were repeated at least three times, and the results were analyzed by two-way ANOVA and presented in GraphPad.

### 2.6. Western Blot Analysis

Protein levels of Fzf1 and a ZF5 deletion variant Fzf1-∆ZF5 in yeast cells were assessed by Western blot analysis. Fzf1-∆ZF5-Flag was constructed into plasmid YCplac111 [27] using C-terminally tagged 3xHA, 3xFlag, and His_6_ (YCpL-*FZF1*-HFH) as a template by PCR amplification using overlapping truncation primers (Table S2) followed by *Dpn*I digestion [28]. To make N-terminally tagged constructs, Primer pairs (Table S2) encoding 3xFlag were used for the QuickChange reaction to be inserted at the 5′ ORF of *FZF1* in plasmid YCpL-*FZF1* to form YCPl-3Flag-*FZF1*, followed by site-directed mutagenesis as previously described to introduce desired mutations. To clone these mutations into a multi-copy plasmid, the entire *FZF1* ORF with its native promoter, terminator, and 3xFlag coding sequences was cloned into YEplac181 [27]. Total yeast proteins were extracted using a glass bead method [29], and tagged proteins were detected using an anti-Flag antibody (Sigma, Oakville, ON, Canada, Cat. F1804, 1:5000 dilution). An anti-Pgk1 polyclonal antibody received from Dr. W. Li (Institute of Zoology, Chinese Academy of Sciences) served as an internal control. To quantify relative protein levels, 3xFlag-Fzf1 protein band intensity was measured by densitometry and analyzed by an Image J software (v 1.54r).

### 2.7. Yeast Reporter Gene Assays

To investigate the transcription activation ability of Fzf1 and its various domains, desired *FZF1* coding sequences were PCR amplified using different primers (Table S2), all containing a *Bam*HI restriction site. After cleavage by *Bam*HI, these DNA fragments were cloned into the *Bam*HI site of plasmid pGBT9 [30] to be fused in-frame with the Gal4_BD_ coding sequence. The desired plasmids (Table S1) were screened and confirmed by DNA sequencing of the entire coding region and transformed into a yeast two-hybrid strain PJ69-4a (*MATa trp1-901 leu2-3,112 ura3-52 his3-200 gal4∆ gal80∆ MET2::P_GAL7-_lacZ::P_GAL1-_HIS3a::P_GAL2_-ADE2)* [31]. The transformants growing on the SD-Trp plate were spotted to SD-Trp-His plus various amounts of 3-amino-1, 2, 4-triazole (3AT, Sigma A8056) to assess the fusion gene’s ability to activate the *P_GAL1_-HIS3* reporter gene. Alternatively, transformants growing on SD-Trp plates were used to inoculate liquid SD-Trp medium. An overnight culture was diluted into fresh selective medium to OD_600_ = 0.2 and cultured for another 2 h, followed by chemical treatments as described above. A β-galactosidase (β-Gal) assay was performed as described using ortho-nitrophenyl-β-galactoside (ONPG) as a substrate and the enzymatic product yield was measured at OD_420 nm_ [32].

### 2.8. Recombinant Fzf1-N117 Protein Production and Purification

To make a C-terminal truncation, a pair of deletion primers Fzf1-N117F/N117R (Table S2) was used to perform QuickChange mutagenesis with plasmid pGEX-*FZF1* [3] as the template. The resulting plasmid, pGEX-*fzf1-N117*, was transformed into *E. coli* Rosetta cells and the recombinant protein was induced by 0.1 mM isopropyl β-D-1-thiogalactopyranoside (IPTG). The bacterial cells were then lysed, and the DNA and RNA contaminants were digested using nuclease benzonase (Thermo Scientific, Mississauga, ON, Canada, Cat. 11442587). The protein was subsequently subjected to glutathione sepharose 4B beads affinity purification (Cytiva, Vancouver, BC, Canada, Cat. 45-002-065). The N-terminal GST tag was then removed by PreScission protease, and the cleaved protein was further purified via an additional round of glutathione sepharose beads to remove GST-tagged PreScission protease, followed by the final purification step with heparin beads (Heparin Sepharose™ 6 Fast Flow, Cytiva, Vancouver, BC, Canada, Cat. 17099801). The purity and concentration of the resulting Fzf1-N117 protein were assessed prior to applications.

### 2.9. Electrophoresis Mobility Shift Assay (EMSA)

Each EMSA reaction contained 0.1 pmol of double-stranded DNA (dsDNA) probe, annealed by one fluorescein isothiocyanate (FITC)-labeled strand and one unlabeled complementary strand (Table S2), 2 ng of bovine serum albumin (BSA), 0.5 µg of poly dI-dC (Thermo Scientific, Mississauga, ON, Canada, Cat. 11430605), dissolved in the EMSA binding buffer. The DNA probe and protein were incubated for 20 min on ice prior to adding poly dI-dC. The macromolecules were then resolved in a 6% native polyacrylamide gel and visualized by detecting the FITC fluorescence at 488 nm.

To determine the dissociation constant (Kd), the EMSA band intensities were quantified using Adobe Photoshop by measuring both the shifted (protein–DNA complex) and unshifted (free probe) bands. The binding percentage was calculated as previously described [3]:

Binding (%) = [Shifted Band Intensity/(Shifted + Free Band Intensity)] × 100%.

The binding saturation point was generated by the relative binding percentage at each concentration, then normalized into the maximum observed binding (saturated binding level):

Normalized Binding (%) = (Observed Binding/Saturated Binding) × 100%.

Binding curves were generated using GraphPad Prism software (version 9.5.0) by fitting the data to the “[Agonist] vs. Normalized Response” equation as described:

Binding (%) = 100 × [Protein molar concentration]/(Kd + [Protein molar concentration]).

## 3. Results

### 3.1. fzf1-ZF5 Is Intragenically Epistatic to fzf1-ZF4

**Figure 1:**
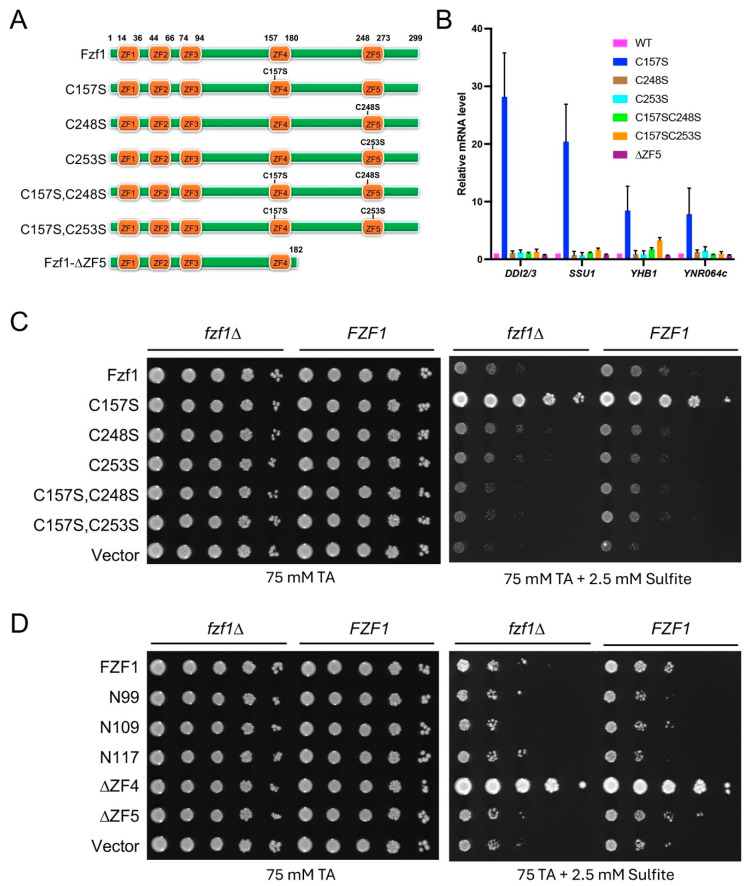
Phenotypes of *fzf1-ZF5* mutants. (**A**) Schematic illustration of different Fzf1 mutations produced by a single-copy YCp plasmid. Amino acid locations of ZF domains, substitutions, and truncation sites are indicated. (**B**) Relative basal-level expression of Fzf1-regulated genes in *fzf1-ZF5* mutants. YCpL plasmids carrying the indicated *fzf1* mutant alleles were transformed into *fzf1*∆ cells, and the transformants were subjected to a qRT-PCR assay. (**C**) Relative sensitivity of *fzf1* amino acid substitution mutants to sodium sulfite. (**D**) Relative sensitivity of *fzf1* truncation mutants to sodium sulfite. (**C**,**D**) YCp plasmids carrying the indicated *fzf1* mutant alleles were transformed into wild-type or *fzf1*∆ cells, and the transformants were subjected to a serial dilution assay. Plates were incubated at 30 °C for three days before photography.

To test a hypothesis that Fzf1-ZF5 serves as a positive regulation domain, we made *fzf1*-*C248S* and *fzf1*-*C253S* mutations and an *fzf1-∆ZF5* truncation in YCpL-*FZF1* [13] by site-directed mutagenesis, as illustrated in Figure 1A, transformed into *fzf1*∆ mutant cells, and measured Fzf1-regulated gene expression. The *DDI2/3*, *SSU1*, *YHB1*, and *YNR064C* transcript levels remained low in wild-type and *fzf1-ZF5* mutants (Figure 1B). Interestingly, under conditions that a *fzf1-ZF4* mutation (*fzf1-C157S*) dramatically increased *DDI2/3*, *SSU1*, *YHB1*, and *YNR064c* basal-level expression, as previously reported [3], *fzf1-C248S* and *fzf1-C253S* point mutations reduced the expression of these genes to levels indistinguishable from that of corresponding *fzf1-ZF5* mutants alone (Figure 1B), indicating that ZF5 is required to support the basal-level expression of Fzf1-regulated genes in the absence of chemical stresses.

Furthermore, plasmids carrying the *fzf1-ZF5* point mutations were transformed into both wild-type and its *fzf1*∆ mutant cells, and their sulfite resistance phenotypes were assessed by a serial dilution assay using *FZF1* and *fzf1-C157S* as references. Figure 1C shows that while the *fzf1-C157S* transformant was highly resistant to sulfite, *FZF1*, *fzf1*-*C248S*, and *fzf1*-*C253S *transformed cells remained sensitive to sulfite, consistent with our observation that the *SSU1* expression was not elevated in these cells (Figure 1B). Interestingly, the *fzf1-C157S*, *C248S* and, *fzf1-C157S*, *C253S* double mutants, displayed phenotypes indistinguishable from those of *fzf1*-*C248S* and *fzf1*-*C253S* single mutants regardless of the presence or absence of a wild-type *FZF1* gene (Figure 1C). We also made various Fzf1 truncations in plasmid YCpL-*FZF1* and found that deletion of ZF4 alone (∆ZF4, missing aa99-182) caused strong sulfite resistance, while deletion of ZF5 alone (∆ZF5, missing aa183-299) or both ZF4 and ZF5 (N99, N109 and N117, containing Fzf1 N-terminal region up to the indicated amino acid residues) remained sensitive to sulfite in both wild-type and *fzf1*∆ backgrounds (Figure 1D). Since *fzf1-ZF4,5* dual mutations and deletions behaved like their corresponding *fzf1-ZF5* single mutants and differed from *fzf1-ZF4* single mutants with respect to both target gene expression (Figure 1B) and sulfite sensitivity (Figure 1C,D), *fzf1-ZF5* mutations are intragenically epistatic to *fzf1-ZF4*, indicating that all *fzf1-ZF5* alleles are loss-of-function mutations.

### 3.2. fzf1-C248S and fzf1-C253S Differentially Affect Fzf1-Regulated Gene Expressions in Response to Chemical Stresses

**Figure 2:**
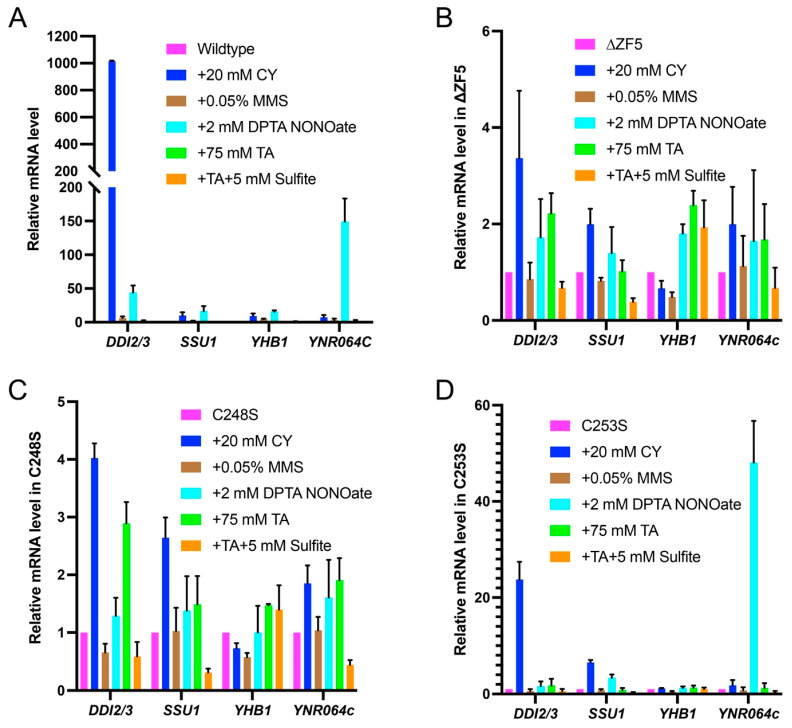
Relative transcript levels of Fzf1-regulated genes in yeast *fzf1-ZF5* mutants under chemical treatment conditions. (**A**) Wild-type *FZF1*. (**B**) *fzf1*-*∆ZF5*. (**C**) *fzf1*-C248S. (**D**) *fzf1*-*C253S*. YCp plasmids carrying the indicated *fzf1* mutant alleles were transformed into BY4741 *fzf1∆* cells, and the transformants were subjected to chemical treatments followed by qRT-PCR assays. All values were relative to the corresponding *fzf1* mutant alleles without chemical treatment (magenta bars). The data are the average of at least three independent experiments, with standard deviations shown as error bars.

If ZF5 is a positive regulatory element within Fzf1, the Fzf1-dependent induction of downstream genes upon chemical treatments is expected to be abolished in these mutants. To test this hypothesis, various chemical treatments were applied to *fzf1*∆ cells harboring a single copy of the *FZF1* derivative. It has been previously reported [3] that such a reconstituted system faithfully represented the endogenous *FZF1* activity, as judged by its downstream gene induction upon chemical treatments. Unlike wild-type cells that responded to chemical treatments with characteristic transcriptional profiles (Figure 2A), *fzf1-∆ZF5 *transformed cells barely responded to various chemical treatments, as evident by their downstream gene expression patterns (Figure 2B). Similarly, the *fzf1-C248S* mutant also displayed compromised transcriptional response to all chemical treatments (Figure 2C) in patterns like that of *fzf1-∆ZF5* (cf. Figure 2B,C), indicating that *fzf1-C248S* is a complete loss-of-function mutation. To our surprise, the *fzf1-C253S* mutant displayed rather different induction patterns, in which *DDI2/3* was still induced more than 20-fold by CY, and *YNR064C* was induced approximately 50-fold by NO (Figure 2D). Hence, *fzf1-C253S* is not a complete *FZF1* loss-of-function mutation. Interestingly, the *fzf1-C253S* mutant phenotype is reminiscent of previously reported *fzf1-ZF4* mutants that, in addition to the increased basal-level gene expression, only *DDI2/3* can be further induced by CY, and *YNR064C* can be further induced by NO [3], albeit at different fold induction levels.

### 3.3. FZF1 Is Dominant over fzf1-ZF5 Mutations

**Figure 3:**
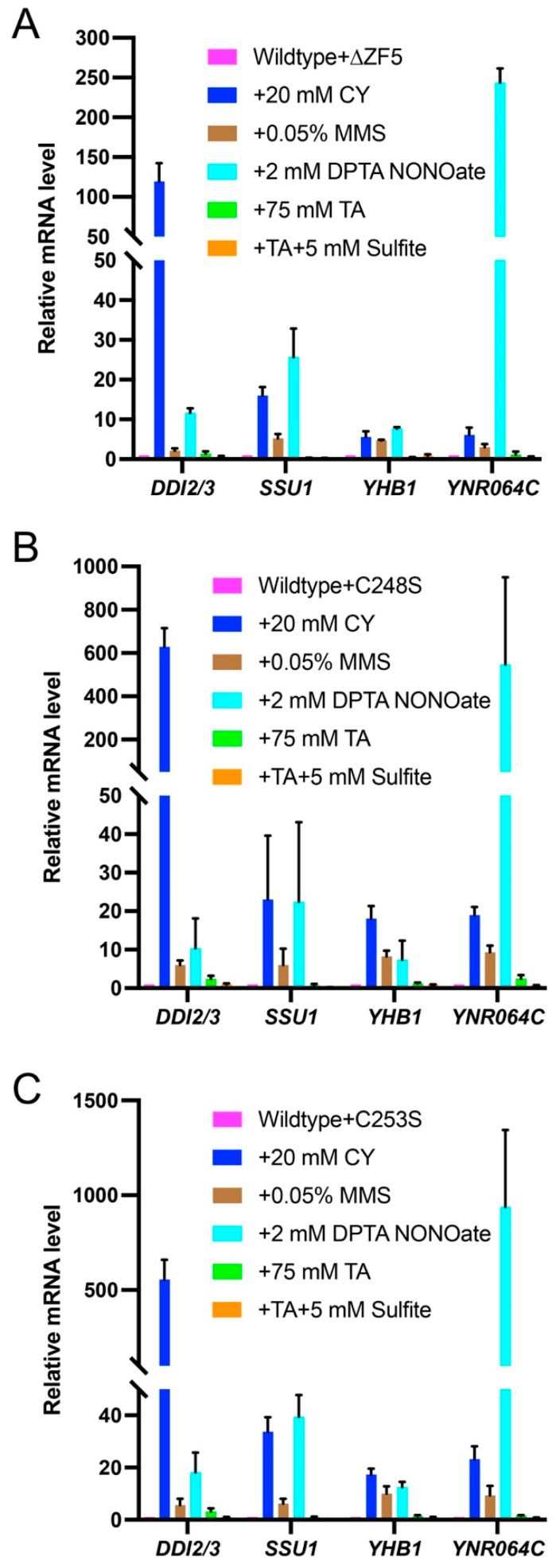
Relative transcript levels of Fzf1-regulated genes in yeast cells carrying both wild-type and *fzf1-ZF5* mutant alleles under chemical treatment conditions. (**A**) *fzf1-∆ZF5*. (**B**) *fzf1*-*C248S*. (**C**) *fzf1*-*C253S*. YCp plasmids carrying the indicated *fzf1* mutant alleles were transformed into BY4741 cells, and the transformants were subjected to chemical treatments followed by qRT-PCR assays. The data are the average of at least three independent experiments, with standard deviations shown as error bars.

If *fzf1-ZF5* is a loss-of-function mutation, it is then expected to be recessive to *FZF1*. To test this hypothesis, YCp plasmids carrying *fzf1-ZF5* mutations were transformed into a wild-type strain so that the transformants carried one copy of *FZF1* on the chromosome, plus one copy of the *fzf1-ZF5* mutant allele in a plasmid. The Fzf1-regulated gene expression was then examined under chemical stress conditions. The wild-type transformant displayed a characteristic response to tested chemicals as previously reported [3], which can also be viewed in Figure 2A. The chemical induction profile in the *fzf1-∆ZF5* transformant in wild-type cells is comparable to that of wild-type cells alone (Figure 2A), except that CY-induced *DDI2/3* expression was reduced by 8.5-fold (Figure 3A, *p* < 0.0001), and the *DDI2/3* induction by CY was reduced by 1.7- and 1.9-fold, respectively, in *fzf1-C248S* (Figure 3B, *p* = 0.0041) and *fzf1-C253S* (Figure 3C, *p* = 0.0014) transformed wild-type cells, while most other gene induction profiles were comparable to wild-type cells. The above observations collectively support the notion that *fzf1-ZF5* mutations are recessive and loss-of-function mutations.

It is noticed that in the presence of *FZF1*, the unique *DDI2/3* induction by CY, as observed in the *fzf1-C253S* mutant (Figure 2D), was undermined (Figure 3C). Meanwhile, NO induced *YNR064C* by nearly 1000-fold in *fzf1-C253S* transformed wild-type cells (Figure 3C), in comparison to approximately 60-fold [3] and 150-fold (Figure 2A) when wild-type cells were growing in rich YPD and minimal SD media, respectively. Furthermore, *fzf1-C248S* (Figure 3B) and empty vector (Supplementary Figure S2) transformed wild-type cells could also support 500-fold induction of *YNR064C* by NO, indicating that this differential induction is mainly due to the combined effects of cell culture media (rich vs. minimal) and genetic compositions (*FZF1* plus different *fzf1* alleles).

### 3.4. fzf1-ZF5 Mutations Are Epistatic to fzf1-ZF4 in Response to Chemical Stresses

**Figure 4:**
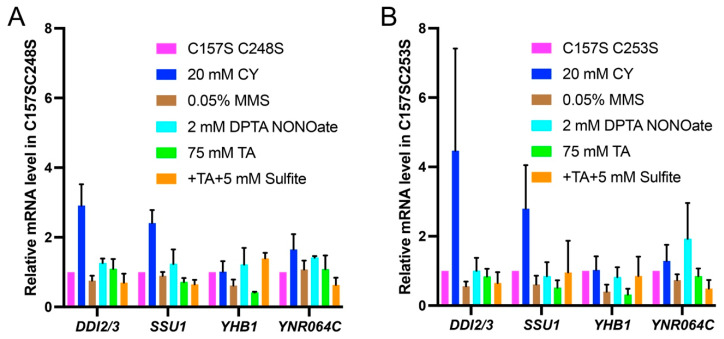
Relative transcript levels of Fzf1-regulated genes in yeast *fzf1-*ZF4,5 double mutants under chemical treatment conditions. (**A**) *fzf1*-*C157S,C248S*. (**B**) *fzf1*-*C157S,C253S*. YCp plasmids carrying the indicated *fzf1* mutant alleles were transformed into BY4741 *fzf1∆* cells, and the transformants were subjected to chemical treatments followed by qRT-PCR assays. The data are the average of at least three independent experiments, with standard deviations shown as error bars.

Since *fzf1-ZF5* mutations abolished the increased basal-level expression of all Fzf1-regulated genes conferred by *fzf1-ZF4* mutations, we asked whether *fzf1-ZF5* mutations could also eliminate chemical induction of these genes in the *fzf1-ZF4* mutant. *fzf1-ZF5* mutations not only reversed basal-level increase, but also additional CY-induced *DDI2/3* and NO-induced *YNR064C* expression found in the *fzf1-C157S* mutant (Figure 4A,B), which allowed us to conclude that *fzf1-ZF5* is epistatic to *fzf1-ZF4* under both spontaneous and chemical-induced conditions. To our surprise, despite that *DDI2/3* and *YNR064C* were inducible by CY and NO, respectively, in both *fzf1-C157S* [3] and *fzf1-C253S* mutant cells (Figure 2D), the *fzf1*-*C157S,* *C253S* double mutation abolished their induction (Figure 4B).

**Figure 5:**
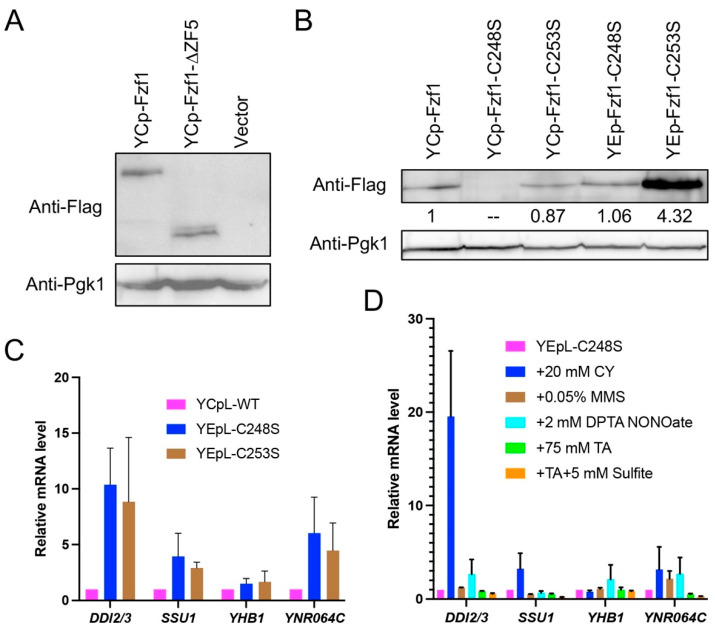
Assessment of cellular Fzf1-derived protein levels. (**A**) C-terminally 3xFlag-tagged Fzf1-∆ZF5 protein. (**B**) N-terminally 3xFlag-tagged Fzf1-C248S and Fzf1-253S proteins. Values are relative to YCp-Fzf1 transformants as measured by densitometry, normalized to the corresponding Pgk1 level. (**A**,**B**) YCp or YEp plasmids carrying wild-type or indicated *fzf1*-*∆ZF5* mutant allele fused to a 3xFlag tag were transformed into BY4741 *fzf1*∆ mutant cells, and the transformants were subjected to Western blot analysis against anti-Flag (upper panel) and anti-Pgk1(lower panel) antibodies. (**C**) Relative transcript levels of Fzf1-regulated genes in the indicated *fzf1* mutant cells. (**D**) Relative transcript levels of Fzf1-regulated genes under chemical treatment conditions. Plasmids carrying indicated *fzf1* mutant alleles were transformed into BY4741 *fzf1∆* cells, and the transformants were untreated (**C**) or treated with indicated chemicals followed by qRT-PCR assays. The data are the average of at least three independent experiments, with standard deviations shown as error bars.

As loss-of-function mutations, one concern was that the mutant form of proteins may not be folded properly and hence affect their cellular levels. We examined in vivo protein levels of C-terminally Flag-tagged single-copy Fzf1 and Fzf1-∆ZF5 under the control of their own promoter and terminator sequences and found that deletion of ZF5 from Fzf1 did not affect its cellular protein level (Figure 5A). While N-terminally Flag-tagged Fzf1-C253S from a single-copy plasmid did not appear to alter its protein stability, the corresponding Fzf1-C248S protein was barely detectable (Figure 5B). Overexpression of the *fzf1-C248S* allele from a multi-copy plasmid displayed a protein level comparable to those of YCpL-*FZF1-* and YCpL-*fzf1-C253S* transformed cells (Figure 5B). Under the above experimental conditions, YEp-*fzf1-C248S* transformed cells moderately increased basal-level expression of Fzf1-regulated genes indistinguishable from that of YEp-*fzf1-C253S* (Figure 5C), despite that the latter produced fourfold more protein (Figure 5B).

We also measured Fzf1-regulated gene expression in response to various chemical stresses in YEp-*fzf1-C248S* transformed *fzf1*∆ cells. As shown in Figure 5D, these genes were not induced by the tested chemical treatments except that *DDI2/3* were induced by CY to nearly 20-fold, reminiscent of the previously reported *fzf1-ZF4* mutant cells [3].

### 3.5. ZF5 Is Dispensable for the Fzf1 Target Sequence CS2 Recognition

**Figure 6:**
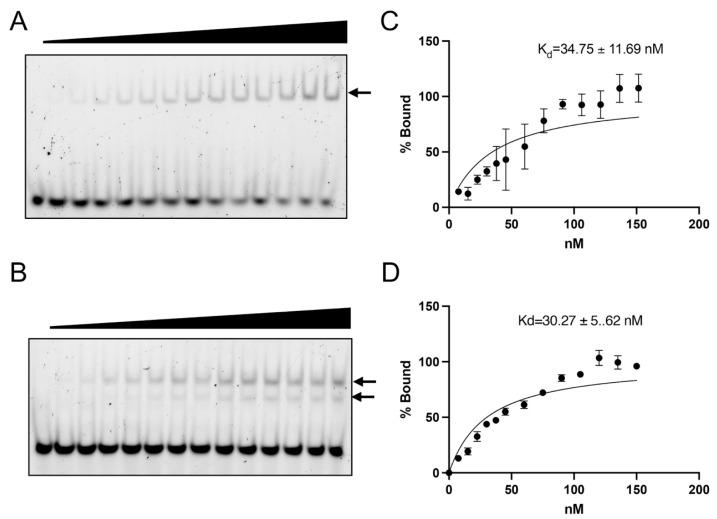
EMSA assessment of sequence-specific interactions of Fzf1 and Fzf1-N117 with an *SSU1*-CS2. (**A**,**B**) Representative EMSA images showing Fzf1 (**A**) or Fzf1-N117 (**B**) interaction with an FITC-labeled *SSU1*-CS2 probe. Triangles on top indicate increasing protein concentrations in lanes 1–14: 0, 7.5, 15, 22.5, 30, 37.5, 45, 60, 75, 90, 105, 120, 135, and 150 nM. Arrows point to anticipated protein-DNA complexes. (**C**,**D**) Quantitative analysis of Fzf1 (**C**) and Fzf1-N117 (**D**) binding affinity for the *SSU1*-CS2 probe. K_d_ values were calculated as described in Materials and Methods based on three independent EMSA images, with standard deviations shown as error bars.

Although it was previously reported that three N-terminal ZFs are sufficient to bind its target sequence in the *SSU1* promoter, that study employed a 192 bp promoter probe, and the Fzf1 protected region was distinct from the newly defined *SSU1*-CS2 sequence [18]. To distinguish whether ZF5 acts as a transcription regulatory element or is required for the target DNA recognition, both of which are consistent with the observed *fzf1-ZF5* mutant phenotypes so far, we conducted an EMSA to measure the in vitro DNA-binding affinity of recombinant Fzf1 and Fzf1-N117, which lacks ZF4 and ZF5, for one of Fzf1 target sequences, *SSU1*-CS2. Recombinant Fzf1 was purified as primarily a single protein band as viewed by SDS-PAGE (Supplementary Figure S3), and it formed a single shifted band with FITC-labeled *SSU1*-SC2 in the EMSA (Figure 6A, arrow). Under the same protein purification conditions, recombinant Fzf1-N117 still contained a few minor bands (Figure S3). Consequently, we normalized the same molar amount of Fzf1 and intact Fzf1-N117 (see Figure S3 legend) in each EMSA reaction with the *SSU1*-CS2 probe and detected a major shifted band (Figure 6B, upper arrow) indicative of Fzf1-N117 binding and a minor shifted band (Figure 6B, lower arrow) that may contain further truncated Fzf1. The calculated dissociation constant (Kd) for the full-length Fzf1 was around 35 nM (Figure 6C), consistent with a previous report [3]. In comparison, the calculated Kd for Fzf1-N117 was around 30 nM (Figure 6D), demonstrating that the deletion of ZF5 does not compromise Fzf1’s ability to interact with its CS2 recognition sequence.

### 3.6. The Fzf1-ZF5 Domain Can Function Independently of the Fzf1 DNA-Binding Domain

**Figure 7:**
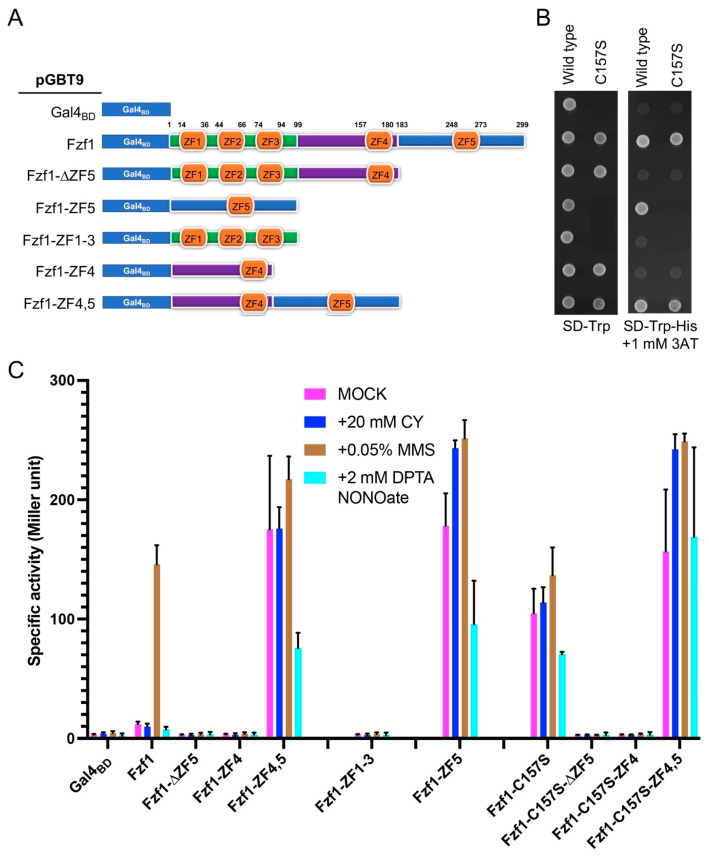
Mapping of the transcription activation domain within Fzf1 by yeast reporter gene assays. (**A**) Schematic illustration of different Fzf1 regions fused to a Gal4 DNA-binding domain (Gal4_BD_) in plasmid vector pGBT9. Amino acid locations of ZF domains and truncation sites are indicated. Plasmid pGBT9 and its *FZF1* fusion constructs were used to transform yeast PJ69-4A cells, and the transformants were subjected to the indicated reporter gene assays. (**B**) A plate-based *P_GAL1_*-*HIS3* reporter assay. The plates were incubated at 30 °C for 2 days before photography. Only one representative plate is shown. (**C**) A *P_GAL7_*-*lacZ* reporter assay that measures β-galactosidase activity. The data are the average of at least three independent experiments, with standard deviations shown as error bars.

Based on the above observations, we hypothesized that Fzf1-ZF5 functions as a TAD independently of its DNA-binding domain. To test this hypothesis, we took advantage of the fact that the majority of transcription activators contain at least two independent domains, namely sequence-specific DNA binding and activation, and that the two domains can often be separated and reconstituted [33,34]. We hence fused various Fzf1 fragments to a Gal4 DNA binding domain (Gal4_BD_) in a yeast two-hybrid vector pGBT9 [30], as shown in Figure 7A, and the resulting plasmids were transformed into a *GAL* reporter strain pJ69-4a [31]. As anticipated, cells producing Gal4_BD_ alone cannot drive the *P_GAL1_-HIS3* reporter gene expression and hence did not grow on the SD-Trp-His + 1 mM 3AT plate. In contrast, cells expressing full-length Fzf1 fused to Gal4_BD_ grew on the above plate (Figure 7B), indicating that Fzf1 contains a TAD. This TAD in Fzf1 was mapped to ZF5 by detailed truncation analysis, as ZF5 is required and sufficient to activate the *P_GAL1_-HIS3* reporter gene when fused to Gal4_BD_ (Figure 7B). Interestingly, Fzf1-ZF4 in the Gal4_BD_ fusion context did not appear to affect the ZF5 activity, nor did the *fzf1-C157S* mutation (Figure 7B).

The above plate-based assay, although informative, could not effectively address two questions. Firstly, it could not tell the relative strength of activation by Fzf1-ZF5 in different contexts. Secondly, one could not ask whether and how Fzf1 and its derivatives respond to different chemical stresses when fused to a heterologous DNA-binding domain. We took advantage that pJ69-4a also harbors a *P_GAL7_-lacZ* reporter and performed a quantitative β-gal assay. As summarized in Figure 7C, when fused to Gal4_BD_, ZF5 alone was sufficient to activate *P_GAL7_-lacZ* by nearly 200-fold, and ZF4 alone did not confer transcription activation, nor did it repress the ZF5 activity. On the other hand, full-length Fzf1 fused to Gal4_BD_ activated *P_GAL7_-lacZ* by more than tenfold, which is sufficient to display a positive result in a plate-based *P_GAL1_-HIS3* reporter assay (Figure 7B), while a C157S substitution further induced *P_GAL7_-lacZ* by another tenfold. In all cases, the chemical treatments did not further induce *P_GAL7_-lacZ* expression, and the NO treatment even slightly reduced already increased basal-level expression. The only exception was that Gal4_BD_-Fzf1 mediated MMS induction of *P_GAL7_-lacZ* by more than tenfold (*p* < 0.0001), whose underlying mechanism remains unclear. These observations collectively demonstrate that the Fzf1-ZF5 domain alone can replace Gal4_AD_ in the heterologous context. In addition, ZF4 serves as a repressor of ZF5 only in the presence of the Fzf1 DNA-binding domain.

## 4. Discussion

This study focused on the functional characterization of ZF5 within the transcription factor Fzf1, and several observations support ZF5 as a stand-alone TAD. Firstly, deletion or point mutations that specifically disrupted the ZF5 domain resulted in loss of Fzf1-mediated chemical induction of its downstream genes. Secondly, the elevated basal-level expression of Fzf1-regulated genes by ZF4 deletion or point mutations is dependent on the intact ZF5 domain. Thirdly, the Fzf1 N-terminal three ZF3 are required and sufficient for the efficient CS2 sequence recognition in vitro, indicating that ZF4 and ZF5 are dispensable for the target DNA-binding activity. Finally, when fused to a Gal4 DNA-binding domain, ZF5 alone functions as a TAD and induces the *P_GAL7_-lacZ* reporter gene expression by nearly 200-fold. It was noted that this strongly elevated activity by ZF5 cannot be further induced by the optimal chemical treatments as observed in Fzf1, indicating that ZF5 serves as a stand-alone TAD.

The Fzf1-C248S protein was found to be unstable in host cells, and hence, interpretation of results related to YCpL-*fzf1-C248S* transformed cells must be cautious. Nevertheless, since both *fzf1-∆ZF5* truncation and the *fzf1-C253S* mutation did not affect cellular protein levels, these results remain sufficient to support our major conclusions in this study. Interestingly, when the cellular Fzf1-C248S protein was brought to a level comparable to Fzf1 by using a multi-copy plasmid, it elevated basal-level expression of Fzf1-targeted genes indistinguishable from that of YEpL-*fzf1-C253S* transformed cells, indicating that Fzf1-C248S and Fzf1-C253S function similarly. Collectively, these two C-to-S substitutions abolished ZF5’s transactivation activity but not the remaining CY-induced *DDI2/3* and NO-induced *YNR064C* expression, which appear to be dependent on Fzf1 but independent of its TAD.

**Figure 8:**
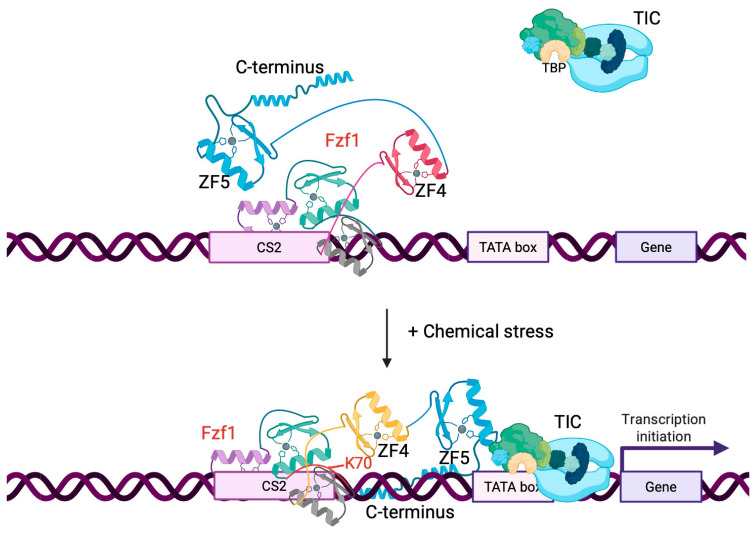
Illustration of a proposed model for Fzf1-mediated transcriptional activation of downstream genes in response to chemical stresses. In this model, the DNA-binding domain consisting of the first three ZFs (ZF1-3) of Fzf1 binds CS2 at its target gene promoters. In the absence of chemical stress, the ZF4 domain interferes with ZF5, preventing it from interacting with the transcription initiation complex (TIC), thereby repressing transcription initiation. Chemical treatments by CY, MMS, or NO cause Fzf1 post-translational modifications (e.g., Lys70 methylation by MMS treatment) that enhance its binding to CS2 and also cause conformational changes to relieve this inhibition, enabling ZF5 to recruit the TIC and ultimately initiate transcription of Fzf1 target genes.

Based on the observed genetic relationships among *FZF1*, *fzf1-ZF4*, and *fzf1-ZF5*, we propose a working model (Figure 8), in which Fzf1 binds its CS2 sequence in the promoters of its regulated genes, including *DDI2/3*, *YHB1*, *SSU1*, and *YNR064C*. However, under uninduced conditions, the ZF5-mediated transcription activation activity is inhibited by ZF4, while the chemical stresses relieve such an inhibition, resulting in ZF5 derepression and the Fzf1 target gene induction. Hence, the chemical stress-induced activation of Fzf1 is at least partially achieved by derepression (Figure 8). Our recent studies [13,14] indicate that chemical stresses like CY and MMS also enhance Fzf1 binding to its target CS2 sequence in vitro and in vivo, although the underlying molecular mechanisms by which chemical stresses relieve ZF4 inhibition remain unclear. It is noted that when fused to Gal4_BD_, the full-length Fzf1 only activated the *P_GAL7_-lacZ* reporter by tenfold, suggesting that the ZF5 activity is still largely inhibited in its native conformation. Indeed, this inhibition can be relieved by a *fzf1-ZF4* mutation. Furthermore, this inhibition can also be relieved by MMS, but not by CY or NO treatment. In this context, it is interesting to note that the purified Fzf1 protein can be methylated by MMS at its K70 residue in vitro, and an *fzf1-K70A* mutation abolishes MMS-induced *DDI2/3* expression [13]. Since K70 is located within the Fzf1 DNA-binding domain, it explains why this domain is required to sense Lys methylation by MMS. In contrast, the Gal4_BD_ belongs to a Zn_2_Cys_6_ family [35] that adopts a structure different from the C_2_H_2_ Fzf1_BD_ and hence may not sense chemical stresses. Like MMS, which can methylate Lys or Arg residues in protein [36], CY can also modify a Lys residue to form homoarginine [37], and a Cys residue can be S-nitrosylated by NO [38,39,40] to alter the protein activity. Apparently, CY- and NO-mediated Fzf1 activation mechanisms cannot be recapitulated in the Gal4_BD_ fusion system and must be different from MMS-mediated Fzf1 activation, which requires further investigation. On the other hand, the full-length Fzf1 conformation appears to be required for ZF4 to inhibit the ZF5 activity, since, when fused to Gal4_BD_, an Fzf1 C-terminal peptide containing both ZF4 and ZF5 behaves like ZF5 alone. Similarly, an *fzf1-ZF4* mutation in this fusion construct did not further induce the *P_GAL7_-lacZ* reporter activity, indicating that this C-terminal region either does not contain a chemical sensor, or ZF4 alone is insufficient to repress the ZF5 activity.

TADs typically lack conserved sequence motifs. Nevertheless, certain structural and sequence features have been identified among various TADs, including acidic/nine-amino-acid TAD (9aaTAD), glutamine-rich domains, serine/threonine-rich domains, proline-rich domains, and isoleucine-rich domains [41,42]. For example, the budding yeast Msn2 9aaTAD interacts with Gal11/Med15, a component of the Mediator complex [43,44]. Mammalian Sp1 contains glutamine-rich and serine/threonine-rich regions to facilitate interactions with various transcriptional co-regulators, including TATA-binding protein (TBP), TAF4 (a TFIID subunit), and chromatin-modifying proteins such as the histone acetyltransferase p300 [17,45]. Mammalian CTF/NF-1 contains both proline-rich and isoleucine-rich domains that interact with components of general transcriptional initiation proteins, including TFIIA, TFIIB, TFIID, TFIIE, TFIIF, and other subunits of preinitiation complex (PIC) as well [46,47]. Although we are not aware of a reported case in which a ZF domain serves as a TAD, C_2_H_2_-type ZF domains are known to not only bind DNA and RNA, but also mediate protein–protein interaction [48], making it possible for ZF5 to interact with and recruit general transcriptional factors to the Fzf1 target gene promoters. We speculate that this interaction must be sequence specific, since Fzf1-ZF4 cannot replace the ZF5 activity. In contrast, ZF4 in its native conformation may block such an access until receiving chemical stress signals (Figure 8).

## Data Availability

The original contributions presented in this study are included in the article/Supplementary Material. Further inquiries can be directed to the corresponding author.

## Supplementary Materials

The following supporting information can be downloaded at: https://www.mdpi.com/article/10.3390/jof12010015/s1, Figure S1. The amino acid sequence of Fzf1. This sequence was translated from the sequenced *FZF1* ORF used in this study, which also agrees with the Saccharomyces Genome Database. Five C_2_H_2_ zinc fingers are highlighted and labeled, and critical residues substituted in this study are in green. Figure S2. Relative *YNR064C* transcript levels upon 2 mM DPTA NONOate (NO) treatment. BY4741 wildtype cells were transformed with a YCplac111 empty vector and the transformant was treated with indicated chemical for 1.5 hour followed by a qRT-PCR assay. The data are the average of at least three independent experiments, with standard deviations shown as error bars. Figure S3. SDS-PAGE analysis of recombinant Fzf1 and Fzf1-N117 proteins. Recombinant Fzf1 protein yielded one predominant band, whose protein concentration was measured by Nano-drop and converted to molar value based on its calculated molecular weight. In contrast, the recombinant Fzf1-N117 protein sample contained several minor bands despite repeated attempts of purification. The concentration of the intact Fzf1-N117 band was measured by densitometry in comparison to that of known Fzf1 concentration and then converted to molar value taken into consideration of molecular weight difference. Each lane contains 3.25 μM of the anticipated protein. Molecular weight markers are shown on left. Table S1. Plasmids used in this study. Table S2. Oligonucleotides used in this study.
