# Supplementary material for: Zinc-Finger 5 Is an Activation Domain in the Saccharomyces cerevisiae Stress-Responsive Transcription Factor Fzf1
Source: J Fungi (Basel). 2025 Dec 25;12(1):15. doi: 10.3390/jof12010015 (PMC12843318; doi:10.3390/jof12010015)
Supplement: Supplementary file 1 [file jof-12-00015-s001.zip › jof-3690988-supplementary.pdf]

Supplementary Materials

Table S1. Plasmids used in this study

Plasmid name	Key features
YCplac111	YCp, *LEU2* (Vector control)
YEplac181	YEp, *LEU2* (Vector control)
YCpL-*FZF1*	*FZF1* ORF along with its own promoter and terminator sequences cloned into YCplac111
YCpL-*fzf1-C157S*	C157S mutation made in YCpL-*FZF1*
YCpL-*fzf1-C248S*	C248S mutation made in YCpL-*FZF1*
YCpL-*fzf1-C253S*	C253S mutation made in YCpL-*FZF1*
YCpL-*fzf1-C157S,C248S*	C248S mutation made in YCpL-*fzf1-C157S*
YCpL-*fzf1-C157S,C253S*	C253S mutation made in YCpL-*fzf1-C157S*
YCpL-*fzf1-N99*	YCpL-*FZF1* truncation encoding aa. 1-99 (ZF1-3)
YCpL-*fzf1-N109*	YCpL-*FZF1* truncation encoding aa. 1-109 (ZF1-3)
YCpL-*fzf1-N117*	YCpL-*FZF1* truncation encoding aa. 1-117 (ZF1-3)
YCpL-*fzf1-ΔZF4*	YCpL-*FZF1* truncation lacking aa. 99-182 coding sequence
YCpL-*fzf1-ΔZF5*	YCp-L-*FZF1* truncation encoding aa. 1-182 (ZF1-4)
YCpL-*FZF1*-HFH	YCp-L-*FZF1* with C-terminal 3xHA, 3xFlag and His_6 tags
YCpL-*fzf1-ΔZF5*-HFH	YCp-L-*fzf1ΔZF5* with C-terminal 3xHA, 3xFlag and His_6 tags
YCpL-Flag-*FZF1*	YCp-L-*FZF1* with an N-terminal 3xFlag tag
YCpL-Flag-*fzf11-C248S*	YCpL-*fzf1-C248S* with an N-terminal 3xFlag tag
YCpL-Flag-*fzf11-C248S*	YCpL-*fzf1-C253S* with an N-terminal 3xFlag tag
YEpL-Flag-*fzf11-C248S*	YEpL-*fzf1-C248S* with an N-terminal 3xFlag tag
YEpL-Flag-*fzf11-C248S*	YEpL-*fzf1-C253S* with an N-terminal 3xFlag tag
pGBT9	YEp, *TRP1*, P_{ADH1}, T_{CYC1}, $Gal4_{BD}$
pGBT-*FZF1*	*FZF1* ORF cloned into pGBT9
pGBT-*fzf1-ΔZF5*	pGBT-*FZF1* truncation encoding aa. 1-182 (ZF1-4)
pGBT-*fzf1-ZF5*	pGBT-*FZF1* truncation encoding aa.183-299 (ZF5)
pGBT-*fzf1-ZF1-3*	pGBT-*FZF1* truncation encoding aa. 1-99 (ZF1-3)
pGBT-*fzf1-ZF4*	pGBT-*FZF1* truncation encoding aa. 99-182 (ZF4)
pGBT-*fzf1-ZF4,5*	pGBT-*FZF1* truncation encoding aa. 99-299 (ZF4-5)
pGEX-6p-1	Amp^R, P_{tac}-MCS-GST, *lacI*^{q}, PreScission protease cleavage site
pGEX-*FZF1*	*FZF1* ORF cloned into pGEX-6p-1
pGEX-*fzf1-N117*	pGEX-*FZF1* truncation encoding aa. 1-117

Table S2. Oligonucleotides used in this study

Name	Sequence (5'-3')
Fzf1-C248S-F	GTCGGATAACCGGAGCAAACATAGTGATTG
Fzf1-C248S-R	CAATCACTATGTTTGCTCCGGTTATCCGAC
Fzf1-C253S-F	CAAACATAGTGATAGTCAAGAGCTTAGTCC
Fzf1-C253S-R	GGACTAAGCTCTTGACTATCACTATGTTTG
Fzf1-N99-F	CATTTAAATAGTCATGAAAGAAAAAGCAAGTGAACGC CATAGAAGAGCAATTTCCGTCCT
Fzf1-N99-R	GAGGACGGAAATTGCTCTTCTATGGCGTTCACTTGCTT TTTCTTTCATGACTATTTAAATG
Fzf1-N109-F	CTTGCATCAAGAATTGACCGTAAACACGAATGAACGC CATAGAAGAGCAATTTCCGTCCT
Fzf1-N109-R	GAGGACGGAAATTGCTCTTCTATGGCGTTCATTCGTGT TTACGGTCAATTCTTGATGCAAG
Fzf1-N117-F	CACGAAGGAGTGAATGCGAATGTGAAAGCATGAACG CCATAGAAGAGCAATTTCCGTCCT
Fzf1-N117-R	GAGGACGGAAATTGCTCTTCTATGGCGTTCATGCTTTC ACATTCGCATTCACTCCTTCGTG
Fzf1-ΔZF5-F	ATAAATCATATGTTGCAACATCATATAGCATGAACGC CATAGAAGAGCAATTTCCGTCCT
Fzf1-ΔZF5-R	AGGACGGAAATTGCTCTTCTATGGCGTTCATGCTATAT GATGTTGCAACATATGATTTAT
Fzf1-ΔZF5-Flag-F	ATAAATCATATGTTGCAACATCATATAGCAGGTACCG GATCCATGGCTTACCCATACGAT
Fzf1-ΔZF5-Flag-R	CATCGTATGGGTAAGCCATGGATCCGGTACCTGCTAT ATGATGTTGCAACATATGATTTA
SSU1-CS2-F	CTTCCTGCAAACTATCATTTTTTTTTC
SSU1-CS2-R	GAAAAAAAAATGATAGTTTGCAGGAAG
$Gal4_{BD}$-BamHI-Fzf1-full-F	CGGGATCCGTATGACGGATATAGGG
$Gal4_{BD}$-BamHI-Fzf1-ZF4-F	CGGGATCCGTAAGCTTGCATCAAGA

$Gal4_{BD}$-BamHI-Fzf1-ZF5-F	CGGGATCCGTAGTAAGCTTGTTGTACCA
$Gal4_{BD}$-BamHI-Fzf1-full-R	AACTGCAGGTCAGTATTCGAATAA
$Gal4_{BD}$-BamHI-Fzf1-ZF4-R	AACTGCAGGTGCTATATGATGTTGCAA
$Gal4_{BD}$-BamHI-Fzf1-ZF1-3-R	AACTGCAGGGCTTTTTCTTTCATGACT
YCp-3Flag-Fzf1-F1	ACATGCATGCGATCAAAGAGATGGTGGCCA
YCp-3Flag-Fzf1-R1	GATGTCATGATCCTTGTAATCACCGTCATGGTCCTTGT AGTCCATTGTTTTGGTGGCCAC
YCp-3Flag-Fzf1-F2	TACAAGGATCATGACATCGACTACAAGGATGACGATG ACAAGATGACGGATATAGGGAGA
YCp-3Flag-Fzf1-R2	AAAACTGCAGCTTCGATTTTTTTGTGCCGT

Supplementary figure legends

Figure S1. The amino acid sequence of Fzf1. This sequence was translated from the sequenced *FZF1* ORF used in this study, which also agrees with the Saccharomyces Genome Database. Five C_2H_2 zinc fingers are highlighted and labeled, and critical residues substituted in this study are in green.

Figure S2. Relative *YNR064C* transcript levels upon 2 mM DPTA NONOate (NO) treatment. BY4741 wildtype cells were transformed with a YCplac111 empty vector and the transformant was treated with indicated chemical for 1.5 hour followed by a qRT-PCR assay. The data are the average of at least three independent experiments, with standard deviations shown as error bars.

Figure S3. SDS-PAGE analysis of recombinant Fzf1 and Fzf1-N117 proteins. Recombinant Fzf1 protein yielded one predominant band, whose protein concentration was measured by Nano-drop and converted to molar value based on its calculated molecular weight. In contrast, the recombinant Fzf1-N117 protein sample contained several minor bands despite repeated attempts of purification. The concentration of the intact Fzf1-N117 band was measured by densitometry in comparison to that of known Fzf1 concentration and then converted to molar value taken into consideration of molecular weight difference. Each lane contains 3.25 µM of the anticipated protein. Molecular weight markers are shown on left.

```
                                            ZF1
  1 MTDIGRTKSR NYKCSFDGCE KVYNRPSLLQ QHQNSHTNQK PYHCDEPGCG
       ZF2                                ZF3
 51 KKFIRPCHLR VHKWTHSQIK PKACTLCQKR FVTNQQLRRH LNSHERKSKL

101 ASRIDRKHEG VNANVKAELN GKEGGFDPKL PSGSPMCGEE FSQGHLPGYD
                       ZF4
151 DMQVLQCPYK SCQKVTSFND DLINHMLQHH IASKLVVPSG DPSLKESLPT

201 SEKSSSTDTT SIPQLSFSTT GTSSSESVDS TTAQTPTDPE SYWSDNRCKH
              ZF5
251 SDCQELSPFA SVFDLIDHYD HTHAFIPETL VKYSYIHLYK PSVWDLFEY
```

Figure S1

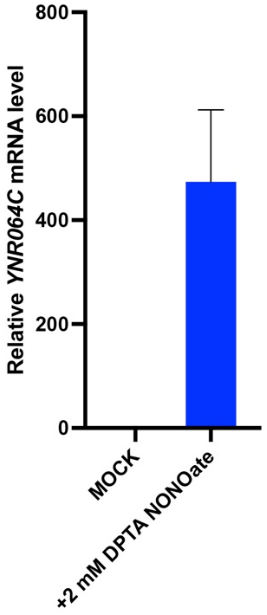
800
600
400
200
0
Relative YNR064C mRNA level
MOCK
+2 mM DPTA NONOate

Figure S2

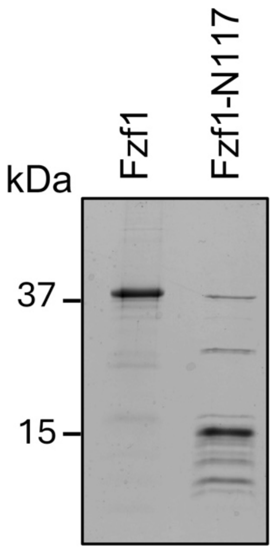

Figure S3
